# Pileup of Insufficient Sleep and Day-to-Day Trajectories of Affective and Physical Well-Being

**DOI:** 10.1093/geroni/igab046.431

**Published:** 2021-12-17

**Authors:** Soomi Lee

**Affiliations:** University of South Florida, Tampa, Florida, United States

## Abstract

This study examined whether and how pileup of insufficient sleep is associated with day-to-day trajectories of affective and physical well-being. Participants from the Midlife in the United States Study (N=1,795) provided diary data for eight days. Pileup of insufficient sleep was operationalized as the number of consecutive nights with <6 hours of sleep. Multilevel models evaluated the linear, quadratic, and cubic effects of pileup of insufficient sleep on daily well-being, adjusting for sociodemographic covariates. Daily negative affect increased and positive affect decreased in curvilinear fashion as the pileup of insufficient sleep increased. For example, daily negative affect increased, but the rate of increase decelerated as the pileup of insufficient sleep increased. In the days most distal to baseline, the rate of increase in negative affect accelerated again. Results were consistent for physical symptoms. Findings suggest that making efforts to break the vicious cycle of insufficient sleep may protect daily well-being.

